# Unintended consequences: Alcohol screening at urban Aboriginal Community Controlled Health Services was suppressed during COVID‐19 lockdowns

**DOI:** 10.1111/dar.13761

**Published:** 2023-10-22

**Authors:** James H. Conigrave, Emma K. Devine, K. S. Kylie Lee, Timothy Dobbins, Julia Vnuk, Noel Hayman, Katherine Conigrave

**Affiliations:** ^1^ Faculty of Medicine and Health, Central Clinical School The University of Sydney Sydney Australia; ^2^ Centre of Research Excellence in Indigenous Health and Alcohol The University of Sydney Sydney Australia; ^3^ The Edith Collins Centre (Translational Research in Alcohol, Drugs and Toxicology), Sydney Local Health District Sydney Australia; ^4^ Institute for Positive Psychology and Education, Australian Catholic University Sydney Australia; ^5^ The Matilda Centre for Research in Mental Health and Substance Use, Faculty of Medicine and Health The University of Sydney Sydney Australia; ^6^ Centre for Alcohol Policy Research La Trobe University Melbourne Australia; ^7^ National Drug Research Institute, Faculty of Health Sciences, Curtin University Perth Australia; ^8^ Burnet Institute Melbourne Australia; ^9^ School of Population Health, UNSW Sydney Sydney Australia; ^10^ Aboriginal Health Council of South Australia Adelaide Australia; ^11^ Adelaide Rural Clinical School The University of Adelaide Adelaide Australia; ^12^ Southern Queensland Centre of Excellence in Aboriginal and Torres Strait Islander Primary Health Care Brisbane Australia; ^13^ School of Medicine University of Queensland Brisbane Australia; ^14^ School of Medicine Griffith University, Gold Coast Campus Gold Coast Australia; ^15^ Drug Health Services Royal Prince Alfred Hospital Sydney Australia

**Keywords:** ACCHSs, alcohol screening, AUDIT‐C, COVID‐19 pandemic, indigenous Australians

## Abstract

**Introduction:**

Regular screening for risky drinking is important to improve the health of Aboriginal and Torres Strait Islander Australians. We explored whether the rate of screening for risky drinking using the Alcohol Use Disorders Identification Test—Consumption (AUDIT‐C) questions was disrupted at Aboriginal Community Controlled Health Services (ACCHS) during state‐wide and territory‐wide COVID‐19 lockdowns in 2020.

**Methods:**

Retrospective analysis of screening data from 22 ACCHSs located in New South Wales, the Northern Territory, Queensland, South Australia, Victoria and Western Australia. These services provide holistic and culturally appropriate primary care. A multi‐level Poisson regression, including AR(1) autocorrelation, was used to predict counts of AUDIT‐C screening at ACCHSs.

**Results:**

AUDIT‐C screening was suppressed during state‐wide and territory‐wide lockdowns in 2020 (incident rate ratio [IRR] 0.42 [0.29, 0.61]). The effect of lockdowns differed by service remoteness. While there was a substantial reduction in AUDIT‐C screening for urban and inner regional services (IRR 0.25 [95% confidence interval (CI) 0.15, 0.42]), there was not a statistically significant change in screening at outer regional and remote (IRR 0.60 [95% CI 0.33, 1.09]) or very remote services (IRR 0.67 [95% CI 0.40, 1.11]).

**Discussion and Conclusions:**

The COVID‐19 lockdowns in Australia likely suppressed rates of screening for risky drinking in urban and inner regional regions. As harm from alcohol consumption may have increased during lockdowns, policymakers should consider implementing measures to enable screening for risky drinking to continue during future lockdowns.

## 1 INTRODUCTION

COVID‐19 ‘lockdowns’ helped curb virus transmission before vaccine development [1]. However, lockdowns can have unintended consequences which must be managed [2]. Lockdowns likely disrupted access to prevention and treatment opportunities for some health conditions [3]. In this report, we examine the effects of lockdowns on screening for risky drinking (drinking that could harm health) at Aboriginal Community Controlled Health Services (ACCHS) in Australia.

Globally, alcohol is the leading cause of mortality among individuals aged 15–49 years [4]. COVID‐19 containment policies, which restricted the ability of individuals to move freely—‘lockdowns’—may have disrupted prevention and treatment pathways [2]. This is particularly problematic for populations that experience greater harm from alcohol. Aboriginal and Torres Strait Islander Australians have been disadvantaged by historical and ongoing harms from British colonisation [5]. Such disadvantages include poorer average health, life expectancy [5] and increased risk of mental health and substance use disorders. Of the preventable risk factors among Aboriginal and Torres Strait Islander Australians, alcohol consumption is the second‐greatest contributor to disease burden [6]. Screening for risky drinking is key to ensuring access to support/treatment for at‐risk Aboriginal and Torres Strait Islander Australians.

ACCHSs offer comprehensive and culturally appropriate primary health care [7]. For many Aboriginal and Torres Strait Islander Australians, ACCHSs—which are locally available and specialised to deliver holistic, culturally appropriate care—are best placed to provide screening for risky drinking and offer treatments when needed [8]. Given the established role of ACCHSs in performing preventive healthcare for Aboriginal and Torres Strait Islander Australians [7], any restriction in their ability to offer screening services should be considered when crafting lockdown policies. However, to date, no study has examined how lockdowns affected the operations of these key services.

In this study, we aimed to determine whether screening for risky drinking was suppressed at ACCHSs during state‐wide COVID‐19 lockdowns. We did this by performing a secondary analysis of data collected for a cluster‐randomised trial.

## 2 METHODS

### 2.1 Study design

We performed a secondary analysis of data from a cluster‐randomised controlled trial that tested whether training and support improve screening and brief intervention rates at ACCHSs [9, 10, 11]. This study received ethical approval from eight committees (see Supporting Information). Data belongs jointly to the 22 participating ACCHSs and The University of Sydney. For enquiries, contact Katherine Conigrave. The intervention, while originally efficacious [9], had no detectable effect during the current study's reference period (6 January 2019–2 January 2021). Here, we explore whether screening rates for risky drinking were reduced during state‐wide COVID‐19 lockdowns.

### 2.2 Dataset and variables

The outcome variable was counts of Alcohol Use Disorders Identification Test—Consumption (AUDIT‐C) questions [12] screens conducted per week. We aggregated data to describe counts of weekly AUDIT‐C screens per service. One ACCHS stopped providing data during the reference period and was excluded. The data describes a clustered time series (one for each of the 21 remaining services). Using a binary variable, we flagged when observations occurred during lockdowns based on date and service location. We only included state/territory‐wide lockdowns for regions where our services were located. Supporting Information includes details about the lockdown dates used (Table S1). We determined a lockdown as having been lifted when citizens could be seated in cafes or restaurants. We included service remoteness [13] as a predictor variable, coded into three levels: ‘Urban and inner regional’, ‘Outer regional and remote’ and ‘Very remote’.

### 2.3 Analysis

We fitted a Poisson zero‐inflated generalised linear mixed mode Sl using the glmmTMB package [14] in R [15]. The AR(1) autocorrelation structure accounted for temporal clustering within services. We predicted AUDIT‐C screening by whether observations occurred during a lockdown. In subsequent models, we included service remoteness as a control variable and then an interaction between lockdown and service remoteness. In each model, we included random intercepts for services and random slopes for lockdown by service.

## 3 RESULTS

A total of 2163 observations of weekly counts of AUDIT‐C screening were collected from 6 January 2019 to 2 January 2021. See Figure S1, Supporting Information, for AUDIT‐C screening for services by state or territory over time. Generally, screening was stable, apart from reduced screening activity in the first week of each year (corresponding with seasonal shutdowns). In each state or territory, there was a marked decrease in screening at the start of the lockdown, which attenuated after the end of the lockdown.

We tested whether the rate of screening was lower during lockdown periods with a Poisson zero‐inflated generalised linear mixed model. The fixed effects of the three models are presented in Table 1. The baseline model (Model 1) predicted that services typically recorded 12.31 [95% confidence interval (CI) 8.20, 18.49] AUDIT‐C screens per week. During COVID‐19 lockdowns, AUDIT‐C screens were substantially reduced incident rate ratio (IRR) = 0.42 [95% CI 0.29, 0.61]. Controlling for service remoteness (Model 2) did not significantly improve model fit. However, adding an interaction between service remoteness and lockdown significantly improved model fit *χ* ^2^(2) = 7.89, *p* = 0.019 (Model 3).

**TABLE 1:** Alcohol Use Disorders Identification Test—Consumption screens per week predicted by COVID‐19 lockdown.

Term	Fixed effects					Likelihood ratio test
	IRR [95% CI]	lnIRR	SE	*z*	*p* value	
Model 1						
Intercept	12.31 [8.20, 18.49]	2.51	0.21	12.10	<0.001	
Lockdown	0.42 [0.29, 0.61]	−0.86	0.19	−4.58	<0.001	
Model 2						*χ* ^2^(2) = 0.78, *p* = 0.677
Intercept	11.21 [5.60, 22.46]	2.42	0.35	6.82	<0.001	
Lockdown	0.43 [0.29, 0.61]	−0.85	0.19	−4.56	<0.001	
Outer regional and remote	0.90 [0.29, 2.86]	−0.10	0.59	−0.17	0.864	
Very remote	1.43 [0.48, 4.29]	0.36	0.56	0.63	0.526	
Model 3						*χ* ^2^(2) = 7.89, *p* = 0.019
Intercept	12.17 [6.60, 22.44]	2.50	0.31	8.00	<0.001	
Lockdown	0.25 [0.15, 0.42]	−1.37	0.26	−5.33	<0.001	
Outer regional and remote	0.79 [0.28, 2.20]	−0.24	0.52	−0.45	0.650	
Very remote	1.23 [0.49, 3.10]	0.21	0.47	0.44	0.663	
Lockdown × Outer regional and remote	2.36 [1.08, 5.16]	0.86	0.40	2.15	0.032	
Lockdown × Very remote	2.64 [1.30, 5.36]	0.97	0.36	2.69	0.007	

*Note*: The model included AR(1) temporal autocorrelation by service, a random slope for the effect of lockdown by services, and random intercepts for services. All models estimated zero inflation by an intercept. Confidence intervals are estimated using the Wald estimation. The IRR coefficient for the intercept represents the baseline number of screens per week. The IRR coefficient for ‘lockdown’ represents the relative increase in screening during weeks that occurred during lockdowns. For Models 2 and 3, the reference level is ‘Urban and inner regional’. *P* values estimated from *z*‐statistic (two‐tailed).

Abbreviations: CI, confidence interval; IRR, incident rate ratio; lnIRR, natural logarithm of the incident rate ratio; SE, standard error of the lnIRR.

The suppression effect of lockdowns was significantly weaker for very remote services compared with services located in urban and inner regional areas. We used the delta method to estimate standard errors for combinations of coefficients, thereby determining the effect of the lockdown for each level of remoteness. During COVID‐19 lockdowns, AUDIT‐C screening was greatly reduced in urban and inner regional services (IRR = 0.25 [0.15, 0.42]), but there was no statistically significant suppression of screening for outer regional and remote (IRR = 0.60 [0.33, 1.09]) or very remote services (IRR = 0.67 [0.40, 1.11]; Table 2).

**TABLE 2:** Relative change in Alcohol Use Disorders Identification Test—Consumption screening due to lockdown by remoteness.

Region	IRR [95% CI]	lnIRR	SE	*z*	*p* value
Urban and inner regional	0.25 [0.15, 0.42]	−1.37	0.26	−5.33	<0.001
Outer regional and remote	0.60 [0.33, 1.09]	−0.51	0.31	−1.69	0.092
Very remote	0.67 [0.40, 1.11]	−0.40	0.26	−1.55	0.121

*Note*: All values are derived from Model 3 in Table 1 using the delta method. Model 3 predicts that the effect of lockdown was significantly different between urban and remote regions. *p* Values estimated from *z*‐statistic (two‐tailed).

Abbreviations: CI, confidence interval; IRR, incident rate ratio; lnIRR, natural logarithm of the incident rate ratio; SE, standard error of the lnIRR.

## 4 DISCUSSION

We aimed to describe how state‐wide and territory‐wide COVID‐19 lockdowns affected AUDIT‐C alcohol screening at ACCHSs. We found that during lockdown periods, AUDIT‐C screening was suppressed by as much as 57.58% across participating ACCHSs. While there was a strong reduction in screening at urban and inner regional services (74.66%), there was no statistically significant change in screening in more remote regions. Aboriginal and Torres Strait Islander Australians are a priority population for preventive health care. At times when lockdowns cannot be avoided, alternative systems should be employed to enable screening (and treatment for clients at risk from drinking when needed).

### 4.1 Comparison to previous findings

Our findings are comparable to studies that showed lockdowns suppressed screening for cancer and sexually transmitted infections by more than half [3, 16]. Our findings add to this literature, showing that alcohol screening was also affected and that suppression effects varied by service remoteness. Urban centres may have been affected more during lockdown periods as COVID‐19 was more prevalent in regions with higher population density. While lockdowns did not prevent people from attending medical appointments, service provision was triaged based on need and staff availability. During times of high transmission, preventive services may not have been offered or clients may have been unwilling to attend clinics except for unavoidable acute care. Thus, part of the suppressing effect of COVID‐19 lockdowns on screening may reflect anxiety about COVID‐19 infection and workforce availability [17] rather than the direct restrictions of lockdown policies.

### 4.2 Potential solutions

Lockdowns can be necessary to contain outbreaks before available vaccination and treatment [18]. When screening cannot take place in clinics, other modalities should be considered. For example, screening for risky alcohol consumption could be conducted remotely via telehealth [19]. However, resources, training and incentives may be needed to ensure it is prioritised. In addition, internet screening tools and phone applications could enable self‐screening and referral to services [20]. However, ensuring access and uptake of e‐screening remains a challenge, as not everyone is comfortable with, or has access to, the internet [21]. For Aboriginal and Torres Strait Islander Australians, Aboriginal Health Workers/Practitioners may play an important role as they are often members of the communities where they work and know which individuals are more at risk. These Aboriginal staff could check in with vulnerable individuals during lockdowns to ensure those at risk are supported and provided with alcohol screening where culturally appropriate.

### 4.3 Implications

Policymakers need to weigh potential unintended consequences against the benefits of short‐term lockdowns during outbreaks of infectious diseases. Protocols are needed to proactively ensure screening for health risk factors and conditions, including risky drinking, can continue during lockdowns.

### 4.4 Limitations

Our findings may not generalise to all outbreaks and lockdowns, which are highly varied. The features and external influences of ACCHSs are varied; our findings will not apply to all ACCHSs in all regions. We only studied suppression of AUDIT‐C screening at ACCHSs, so we cannot rule out that AUDIT‐C screening was replaced by other kinds of screening for risky drinking. We could not separate reduced screening from reduced attendance with our data. Our data does not allow us to estimate the health effects of the reduction in alcohol screening.

## 5 CONCLUSION

Screening for risky alcohol consumption using AUDIT‐C was suppressed at ACCHSs in urban and inner regional settings during the 2020 state‐wide and territory‐wide COVID‐19 lockdowns. Policymakers need to weigh the unintended consequences of lockdowns against benefits. Where possible, strategies to ensure the continuation of screening for modifiable health risk factors should be deployed during future lockdowns.

## AUTHOR CONTRIBUTIONS

KC, KL, TD and NH conceptualised the study and secured funding for it. KC, KL, TD and JC participated in dataset collection. JC curated the dataset. JC, TD and KC devised the analytic plan. JC conducted the analysis. JC wrote the original draft with assistance from ED and KC. JV and NH aided in interpreting findings within the ACCHS context. All authors participated in revisions, approved and are accountable for the final manuscript.

## CONFLICT OF INTEREST STATEMENT

The authors declare no conflicts of interest.

## ETHICS STATEMENT

Ethical approval was obtained from eight ethics committees. The Aboriginal Health and Medical Research Council of NSW Ethics Committee (NSW; Project 1217/16), Central Australian Human Research Ethics Committee (Project CA‐17‐2842), Human Research Ethics Committee of Northern Territory Department of Health and Menzies School of Health Research (Project 2017–2737), Central Queensland Hospital and Health Service Human Research Ethics Committee (Project 17/QCQ/9), Far North Queensland Human Research Ethics Committee (Project 17/QCH/45–1143), the Aboriginal Health Research Ethics Committee, South Australia (SA; Project 04–16‐694), St Vincent's Hospital Melbourne Human Research Ethics Committee (Project LRR 036/17) and Western Australian Aboriginal Health Ethics Committee (WA; Project 779).

## Supporting information


**DATA S1.** Supporting Information.
