# Supplementary material for: Unintended consequences: Alcohol screening at urban Aboriginal Community Controlled Health Services was suppressed during COVID‐19 lockdowns
Source: Drug Alcohol Rev. 2023 Oct 22;42(7):1633–8. doi: 10.1111/dar.13761 (PMC10946595; doi:10.1111/dar.13761)
Supplement: Supplementary file 1 — DATA S1. Supporting Information. [file DAR-42-1633-s001.docx]

## Supporting Information

## Ethical approval

Ethical approval was obtained from eight ethics committees. The Aboriginal Health and Medical Research Council of NSW Ethics Committee (NSW; project 1217/16), Central Australian Human Research Ethics Committee (project CA-17-2842), Human Research Ethics Committee of Northern Territory Department of Health and Menzies School of Health Research (project 2017--2737), Central Queensland Hospital and Health Service Human Research Ethics Committee (project 17/QCQ/9), Far North Queensland Human Research Ethics Committee (project 17/QCH/45--1143), the Aboriginal Health Research Ethics Committee, South Australia (SA; project 04--16-694), St Vincent's Hospital Melbourne Human Research Ethics Committee (project LRR 036/17) and Western Australian Aboriginal Health Ethics Committee (WA; project 779).

## ACCHS recruitment

We identified 132 Aboriginal Community Controlled Health Services (ACCHS) across Australia that were potentially eligible to participate in the broader trial. ACCHSs were identified from a listing on the National Aboriginal Community Controlled Health Organisation website [1]. Services were eligible if they served at least 1,000 unique clients each year. We recruited the first 22 eligible services that were willing to participate. Four services declined to participate. Five services gave no response.

## Service remoteness

To determine service remoteness, used the Australian Standard Geographic Classification system [2]. We coded service remoteness into three levels: “Urban and inner regional”, “Outer regional and remote”, and “Very remote”.

## Aboriginal Community Controlled Health Services

ACCHSs tend to be highly motivated to screen for a wide range of health conditions due to a higher burden of chronic health problems among Aboriginal and Torres Strait Islander Australians [3].

## Lockdown start date and end date

For all the included states and territories, we determined lockdowns to have begun on 23 March 2020 (New South Wales, Northern Territory [4], Queensland [5], South Australia [6], Victoria [7], Western Australia [8]). We determined the lockdown to have ended on 11 May for South Australia [9]; 15 May for New South Wales [10], Northern Territory [11] and Queensland [12]; 18 May for Western Australia [13]; and on 1 June for Victoria [14]. A second state-wide lockdown occurred in Victoria from 5 August [15] to 17 September, 2020 [16]. We focused only on state-wide lockdowns, as these are the lockdowns that our services were affected by. We did not include for example, the Melbourne lockdowns which were particularly long and restrictive, as none of our services were based in Melbourne. We did not include lockdowns affecting smaller regions, as these were less clearly defined and might jeopardise the anonymity of services. Figure S1 visualises the included lockdown periods by state against relative Alcohol Use Disorders Identification Test–Consumption screening rates. Table S1 summarises the lockdown periods and durations.

## COVID-19 context in Australia

In Australia, the first cases of COVID-19 were recorded on 25 January 2020 [17] and by 23 March 2020 containment measures were implemented nationally to slow the spread of the disease [18]. During these ‘lockdowns’ many Australians were only allowed to leave their homes for health care, exercise, shopping for essentials, essential work or essential education. These restrictions remained in place nationally until mid-May 2020 after which restrictions were progressively relaxed [19]. Beyond lockdowns there were continually shifting restrictions placed on Australia which varied by location. While our paper only looks at the effect of state-wide lockdowns, these policies undoubtedly also affected the way clinics operate. For example there were ‘biosecurity zones’ implemented to protect some remote Aboriginal and Torres Strait Islander communities [20]. These zones, e.g., in the Northern Land Councils region restricted the ability of people to enter some Aboriginal and Torres Strait Islander Australian communities [21]. While this prevented the spread of COVID-19, it also would have resulted in staff shortages at clinics as not all workers reside within the communities they serve. In urban centres there were density limits places on businesses which also would have increased community awareness of COVID-19 and altered behaviour. Melbourne also spent more time in lockdown than any other city in the world [22] – none of our services were based in Melbourne, so Melbourne’s lockdowns are not included in our analysis.

Given the constantly changing policy landscape which varied by region, we chose to focus only on the state and national lockdowns which had more definable start and end dates. However, we acknowledge that there were constant changing pressures on each of our services, and lockdowns were only one external influence among many through 2020.

## Comparison to suppression effects at other screening services

Our findings are generally consistent with those studies, which showed that lockdowns suppress screening for a wide range of conditions. Cancer screening in several countries decreased during COVID-19 lockdowns [23], including in Australia [24]. In New South Wales, Australia, breast cancer screening activity reduced by half (51.5%) during the March to June 2020 lockdown period [25]. Similarly, screening for sexually transmitted infections was reduced by more than two-thirds (68%) among asymptomatic patients during Australian COVID-19 lockdowns [26], with some clinics suspending walk-in services and halting screening for asymptomatic individuals [27]. However, it is important to note that screening for cancer and sexually transmitted infections can only be done in person with specialised equipment. Accordingly, these services might be more affected by restrictions in movement than screening services that can be done remotely via telehealth such as alcohol screening.

Table S1. State- and territory-wide lockdowns that occurred in 2020 that affected participating services

| State | Lockdown start | Lockdown end | Duration (days) |
| --- | --- | --- | --- |
| New South Wales | 23 March | 15 May | 53 |
| Northern Territory | 23 March | 15 May | 53 |
| Queensland | 23 March | 15 May | 53 |
| South Australia | 23 March | 11 May | 49 |
| Victoria | 23 March | 1 June | 70 |
| Victoria | 5 August | 17 September | 43 |
| Western Australia | 23 March | 18 May | 56 |

*Note.* We only included state-wide lockdowns which affected participant services.


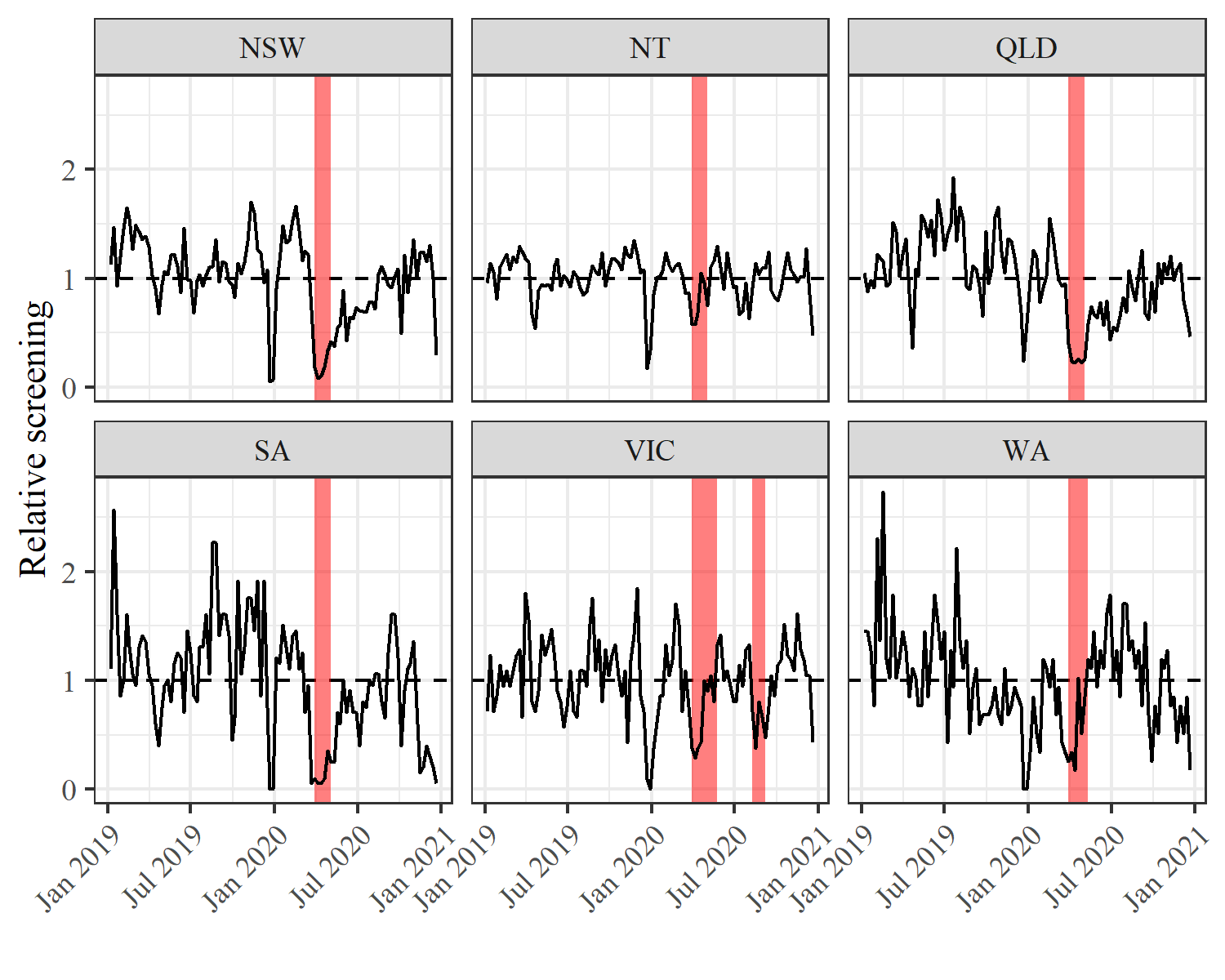

Figure S1. Relative AUDIT-C screening by state. The red shaded regions indicate that a state-wide COVID-19 lockdown was in place during that period. The y-axis shows weekly AUDIT-C screening relative to the median screening rate for a given state.

AUDIT-, Alcohol Use Disorders Identification Test–Consumption.

## References

1. NACCHO. (2016). National Aboriginal Community Controlled Health Organisation website. NACCHO. Available from https://www.naccho.org.au/

2. Australian Bureau of Statistics. (2011). Australian statistical geography standard (ASGS): Volume 5—Remoteness structure. Australian Bureau of Statistics Australia.

3. Gracey M, King M. Indigenous health part 1: determinants and disease patterns. Lancet. 2009;374:65-75.

4. Heggie HC. COVID-19 Directions (No. 6) 2020 (2020). Available from: https://health.nt.gov.au/__data/assets/pdf_file/0008/806840/CHO-Directions-No-6-Closure-of-certain-businesses-and-cease-certain-businesses-SIGNED.pdf

5. Miles S. (23 March 2020). Business closures and restrictions. Ministerial Media Statements. Available from: https://statements.qld.gov.au/statements/89582 (accessed 16 December 2022).

6. Stevens, G. Direction of the state co-ordinator: non-essential business (and other gatherings) closure direction (2020). Available from: https://www.legislation.sa.gov.au/legislation/CV19/non-essential-business-and-other-activities/ceased/Non-Essential-Business-and-Other-Gatherings-Closure-Direction-No-1_23.3.2020_CEASED.pdf

7. Andrews D. (22 March 2020). Statement From The Premier. Available from: http://www.premier.vic.gov.au/statement-premier-61 (accessed 16 December 2022).

8. Government of Western Australia, Premier’s office. (23 March 2020). Important new COVID-19 measures come into effect. Available from: https://www.mediastatements.wa.gov.au/Pages/McGowan/2020/03/Important-new-COVID-19-measures-come-into-effect-.aspx (accessed 16 December 2022)

9. Boseley M, Landis-Hanley J. (20 May 2020). Social distancing rules explained: Australia’s current state by state coronavirus guidelines. Available from: https://www.theguardian.com/australia-news/2020/may/20/social-distancing-rules-australia-when-will-end-guidelines-coronavirus-laws-physical-covid-19-restrictions-signs-posters-nsw-victoria-qld-queensland-act-sa-wa-nt-tasmania (accessed 16 December 2022)

10. NSW Government Digital Channels. (2020). NSW to ease COVID-19 restrictions from Friday 15 May. Available from: https://www.nsw.gov.au/news/nsw-to-ease-covid-19-restrictions-from-friday-15-may

11. Office of the Chief Minister, Northern Territory Government. (18 November 2021). The Territory’s Roadmap to the New Normal. Northern Territory Government Newsroom. Northern Territory Government. Available from: https://newsroom.nt.gov.au/article (accessed 16 December 2022)

12. Palaszczuk A. (8 May 2020). Premier maps road to easing restrictions. Ministerial Media Statements. Available from: https://statements.qld.gov.au/statements/89800 (accessed 16 December 2022)

13. Permier’s office, Government of Western Australia. (10 May 2020). The WA roadmap for easing COVID-19 restrictions. Available from: https://www.mediastatements.wa.gov.au/Pages/McGowan/2020/05/The-WA-roadmap-for-easing-COVID-19-restrictions.aspx (accessed 16 December 2022).

14. Cafes, restaurants and pub dining to resume in Victoria from June. (16 May 2020). ABC News. Available from: https://www.abc.net.au/news/2020-05-17/victoria-coronavirus-cafes-restaurants-pub-dining-to-reopen/12256306

15. Andrews, D. (2 August 2020). Premier’s statement on changes to regional restrictions. Available from: https://www.dhhs.vic.gov.au/updates/coronavirus-covid-19/premiers-statement-changes-regional-restrictions (accessed 17 August 2023).

16. Andrews D. (15 September 2020). Statement from the Premier. Available from: https://www.dhhs.vic.gov.au/updates/coronavirus-covid-19/statement-premier-15-september-2020 (accessed 17 August 2023).

17. Jee Y. WHO International Health Regulations Emergency Committee for the COVID-19 outbreak. Epidemiol Health. 2020;42:e2020013.

18. Morrison S. (20 March 2020). Update on Coronavirus measures [March 2020]. PM Transcripts. Available from: https://pmtranscripts.pmc.gov.au/release/transcript-43967

19. Morrison S. (8 May 2020). Update on Coronavirus measures [May 2020]. PM Transcripts. Available from: https://pmtranscripts.pmc.gov.au/release/transcript-43983

20. Keene M. (30 July 2020). COVID-19 and Indigenous Australians: a chronology. text. Available from: https://www.aph.gov.au/About_Parliament/Parliamentary_Departments/Parliamentary_Library/pubs/rp/rp2021/Chronologies/COVID19-IndigenousAustralians (accessed 29 June 2023)

21. Federal Register of Legislation. (24 April 2020). Biosecurity (Human Biosecurity Emergency) (Human Coronavirus with Pandemic Potential) (Emergency Requirements for Remote Communities) Determination 2020. Attorney-General’s Department. Available from: https://www.legislation.gov.au/Details/F2020C00354/Html/Text, http://www.legislation.gov.au/Details/F2020C00354 (accessed 29 June 2023).

22. Boaz J. Melbourne passes Buenos Aires’ world record for time spent in lockdown. ABC News. 3 October 2021. Retrieved from https://www.abc.net.au/news/2021-10-03/melbourne-longest-lockdown/100510710

23. Mayo M, Potugari B, Bzeih R, Scheidel C, Carrera C, Shellenberger RA. Cancer screening during the COVID-19 pandemic: A systematic review and meta-analysis. Mayo Clin Proc Innov Qual Outcomes. 2021;5:1109-17.

24. Feletto E, Grogan P, Nickson C, Canfell K. How has COVID-19 impacted cancer screening? Adaptation of services and the future outlook in Australia. Public Health Res Pract. 2020;30:3042026

25. Sutherland K, Chessman J, Zhao J, Sara G, Went A, Dyson S, Levesque J-F. Impact of COVID-19 on healthcare activity in NSW, Australia. Public Health Res Pract. 2020;30:3042030

26. Chow EPF, Hocking JS, Ong JJ, Phillips TR, Fairley CK. Sexually transmitted infection diagnoses and access to a sexual health service before and after the national lockdown for COVID-19 in Melbourne, Australia. Open Forum Infect Dis. 2021;8:ofaa536.

27. Phillips TR, Fairley CK, Donovan B, Ong JJ, McNulty A, Marshall L, et al. Sexual health service adaptations to the coronavirus disease 2019 (COVID‐19) pandemic in Australia: a nationwide online survey. Aust N Z J Public Health. 2021;45:622-7.
